# Editorial: Perceived social norms and how they relate to online media

**DOI:** 10.3389/fpsyg.2025.1568119

**Published:** 2025-02-25

**Authors:** Elliot Panek, Saar Mollen, Christopher Cascio

**Affiliations:** ^1^Department of Journalism & Creative Media, University of Alabama, Tuscaloosa, AL, United States; ^2^Faculty of Social and Behavioural Sciences, University of Amsterdam, Amsterdam, Netherlands; ^3^School of Journalism and Mass Communication, University of Wisconsin-Madison, Madison, WI, United States

**Keywords:** social media, social norms, perceived social norms, influence, norms, online

Decades of research on social influence attest to the power of norms to shape a wide range of beliefs, attitudes, and behaviors. Everything from the decision to purchase clothing to deciding to have a child are shaped by one's perceptions of social norms. This is what makes social norms a popular mechanism through which to change behavior (e.g., the social norms approach, Berkowitz, 2005). While neither this phenomenon nor theories that undergird our understanding of it are new, the rapidly evolving social contexts of online life present individuals with new vantage points from which to observe others, and so to form new beliefs about what is normal. In this Research Topic of *Frontiers in Psychology*, we feature work from scholars who examine ways in which perceived social norms are shaped and influence a variety of outcomes.

Injunctive and descriptive social norm appeals are successful tools to motivate behavior. Given the prevalence of normative appeals presented on social media, expanding our understanding of these appeals is important. Two two-wave studies by Schorn and Wirth indicated that though normative appeals influenced perceived norms across both studies, only injunctive majority appeals influenced persuasive outcomes.

In a survey study of perceptions of the benefits of having children and China's birth encouragement policies, Li found a relationship between Chinese women's own attention to social media content and interpersonal communication about the benefits of having children and the presumed influence these messages have on others. This subsequently related to their injunctive and descriptive norm perceptions and personal norms regarding support for birth encouragement policies.

Angelini et al. examined associations between teen interactions on social media with peers and friendship quality. Findings suggest that adolescents who engage in more peer-oriented social media use, value their friendships more. This research highlights the importance of peer norms on social media during adolescence.

Data from a large cross-sectional survey in 17 countries was used to investigate the relationship between distal (similar people in the same country) and proximal (close friends) injunctive norm perceptions and intentions to purchase counterfeit products. Kononova et al. found that distal injunctive norm perceptions predicted proximal injunctive norm perceptions, and proximal norms predicted intentions to make purchases. In countries that score higher on power distance, the relation between distal and proximal injunctive norms was weaker.

The research in this Research Topic has implications for those designing the next generation of social media platforms. Zou et al. find that features intended to increase user engagement by making them more visible to mutual friends may inadvertently curb it. Norm violation online carries the same stigma as it does offline, and the desire to avoid embarrassment, the study suggests, may be more powerful than the desire to connect.

The types of entertainment media we enjoy online are affected by our perceptions of who else enjoys them, as is demonstrated by the research of Wang et al.. Feelings of transportation and immersion in fictional television narratives, the study shows, are partially contingent upon whether one's family and friends recommend it. These findings suggest that there are limits to online streamers' ability to disseminate values across cultures. Enjoyment of narratives is embedded not just in cultural contexts, but in social ones.

This Research Topic highlights how online information and interpersonal communication may work together or even interact to influence perceived social norms, subsequently influencing attitudes, intentions, and behaviors. Social norms are communicated online in various ways (injunctive and descriptive) from various sources (proximal and distal); researchers attempting to understand influence online should account for this variety. Collectively, these studies offer insights into the complex, rapidly evolving social contexts of online life, setting the stage for future research.
